# Convergent evolution in structural elements of proteins investigated using cross profile analysis

**DOI:** 10.1186/1471-2105-13-11

**Published:** 2012-01-16

**Authors:** Kentaro Tomii, Yoshito Sawada, Shinya Honda

**Affiliations:** 1 Computational Biology Research Center (CBRC), National Institute of Advanced Industrial Science and Technology (AIST), 2-4-7 Aomi, Koto Ward, Tokyo 135-0064, Japan; 2 Biomedical Research Institute, National Institute of Advanced Industrial Science and Technology (AIST), AIST Central 6, Tsukuba 305-8566, Japan

## Abstract

**Background:**

Evolutionary relations of similar segments shared by different protein folds remain controversial, even though many examples of such segments have been found. To date, several methods such as those based on the results of structure comparisons, sequence-based classifications, and sequence-based profile-profile comparisons have been applied to identify such protein segments that possess local similarities in both sequence and structure across protein folds. However, to capture more precise sequence-structure relations, no method reported to date combines structure-based profiles, and sequence-based profiles based on evolutionary information. The former are generally regarded as representing the amino acid preferences at each position of a specific conformation of protein segment. They might reflect the nature of ancient short peptide ancestors, using the results of structural classifications of protein segments.

**Results:**

This report describes the development and use of "Cross Profile Analysis" to compare sequence-based profiles and structure-based profiles based on amino acid occurrences at each position within a protein segment cluster. Using systematic cross profile analysis, we found structural clusters of 9-residue and 15-residue segments showing remarkably strong correlation with particular sequence profiles. These correlations reflect structural similarities among constituent segments of both sequence-based and structure-based profiles. We also report previously undetectable sequence-structure patterns that transcend protein family and fold boundaries, and present results of the conformational analysis of the deduced peptide of a segment cluster. These results suggest the existence of ancient short-peptide ancestors.

**Conclusions:**

Cross profile analysis reveals the polyphyletic and convergent evolution of β-hairpin-like structures, which were verified both experimentally and computationally. The results presented here give us new insights into the evolution of short protein segments.

## Background

Abundant examples of similar segments appearing in different protein folds, here continuous structural fragments in native protein folds, have been reported. Although some of those segments are believed to have originated from common ancestors, evolutionary scenarios for many of those segments are not clear. As opposed to the monophyletic scenario of presently existing protein domains, Lupas *et al*. argued the hypothesis of ancient short peptide ancestors [1]. They found local sequence and structure similarities such as P-loops, zinc finger motifs, and Asp boxes, in different protein folds based on results of all-against-all structural comparisons of segments using their rigorous structure comparison method. The reason they employed their structure comparison method is that occurrences of such segments 'might not be expected to be meaningful from a sequence-only perspective [1]'.

Originally, the profile method was developed by Gribskov *et al*. [2]. Since that time, sequence profiles calculated from multiple alignments of protein families have been used for finding distantly related protein sequences. Here, a profile is a table that lists amino acid preferences in each position of a given multiple sequence alignment. Results show that the inclusion of evolutionary information for both the query protein and for proteins in the database being searched improved the detection of related proteins [3]. These profile-profile comparison methods, which are sequence-based methods, are fundamentally superior to the profile method both in their ability to identify related proteins and to improve alignment accuracy [3-5]. Then, Friedberg and Godzik (2005) constructed a segment dataset, called Fragnostic, by combining the scores of their profile-profile comparison method, FFAS03 [6], and the *C*_α _root mean square deviation (RMSD) of the structural alignment. They presented an alternative view of the protein structure universe in terms of the relations between interfold similarity and functional similarity of proteins via segments [7]. They found functional commonalities of proteins with different folds that share the similar segments, such as dimetal binding loops. Therefore, the segments are shared by many different protein folds.

Profile-profile comparison methods have been developed and used for various purposes other than the original one. For instance, profile-profile comparison methods were applied in an attempt to establish evolutionary relations within protein superfolds [8]. In this attempt, among three small β-barrel folds, intra-fold similarity scores calculated using profile-profile comparisons were used to identify functionally distinct sub-families. An amino acid sequence-order-independent profile-profile comparison method (SOIPPA) has been proposed and used for functional site comparison to find distant evolutionary relations by integrating local structural information [9]. Some novel evolutionary relations across folds were detected automatically using SOIPPA. Recently, Remmert *et al*. proposed the possibility of divergent evolution of outer membrane β proteins from an ancestral ββ hairpin using their HMM-HMM comparison method [10]. Using two atypical proteins as analogous reference structures, they argued that similarities of outer membrane β proteins are unlikely to be the result of sequence convergence.

However, no application of profile-profile comparison methods combines sequence-based profiles and structure-based profiles to capture more precise sequence-structure relations. Amino acid sequence patterns in proteins can be represented as profiles constructed using sequence and/or structural information. On one hand, comparison of sequence-based profiles based on evolutionary information is known to be highly effective for protein fold recognition [11], even when they are constructed without including explicit structural information, which indicates that they might harbor structural information. On the other hand, some amino acid substitution patterns, which reflect the physicochemical constraints of local conformations, are well known to correlate strongly with the protein structure at the local level. Profiles or position-specific amino acid propensities based on local structural classification have been used to study local sequence-structure relations for many years [12]. Moreover, libraries of sequence patterns that correlate well with local structural elements have been constructed [13,14]. Amino acid propensities were analyzed at each position of short protein segments within a structural cluster obtained by structural classification methods [15-18]. Position-specific amino acid propensities in protein segments with two consecutive secondary structure elements have also been investigated to support protein structure prediction [19]. Pei and Grishin effectively combined evolutionary and structural information to improve local structure predictions [20].

Consequently, the aim of this study is to identify properties that are common to both profile types, and to find novel sequence-structure relations. To this end, we developed a method we call "Cross Profile Analysis" to compare structure-based profiles originating from the results of local structural classifications, with sequence-based profiles produced by PSI-BLAST using FORTE, our profile-profile comparison method [21,22]. Using structure-based profiles derived from clusters of segment structures with 9-residue and 15-residue lengths as a starting point, we identified several structure-based profiles that correlate well with sequence-based profiles. These correlations indicate structural similarity between conformations of a segment cluster and the local structures corresponding to the segments of a protein family whose sequence-based profile exhibited strong correlation with a structure-based profile. This report describes previously undetectable sequence-structure patterns that transcend protein superfamily and fold boundaries, especially for segments that contain β-hairpin-like structures, shared by proteins with two distinct folds. Furthermore, through experimental measurements, we demonstrate that a deduced peptide corresponding to the segments, which has been shown to exhibit such sequence-structure correlation, is structurally stable in aqueous solution, suggesting the existence of ancient short peptide ancestors. We discuss the possibility of the convergent evolution of the protein short segments with patterns detected using our cross profile analysis.

## Results and discussion

### Cross Profile Analysis

**Figure 1:**
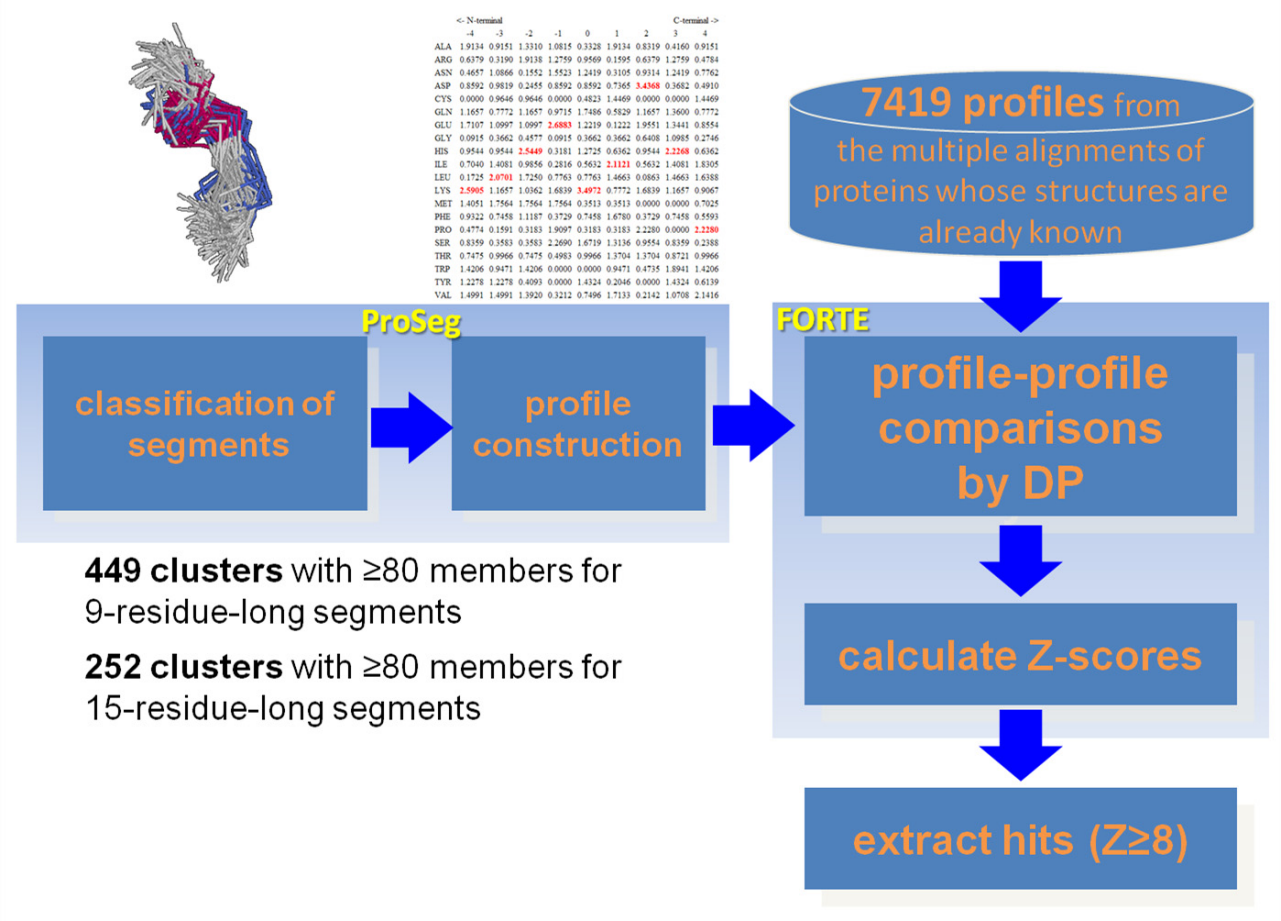
**Schematic representation of cross profile analysis using FORTE**.

**Figure 2:**
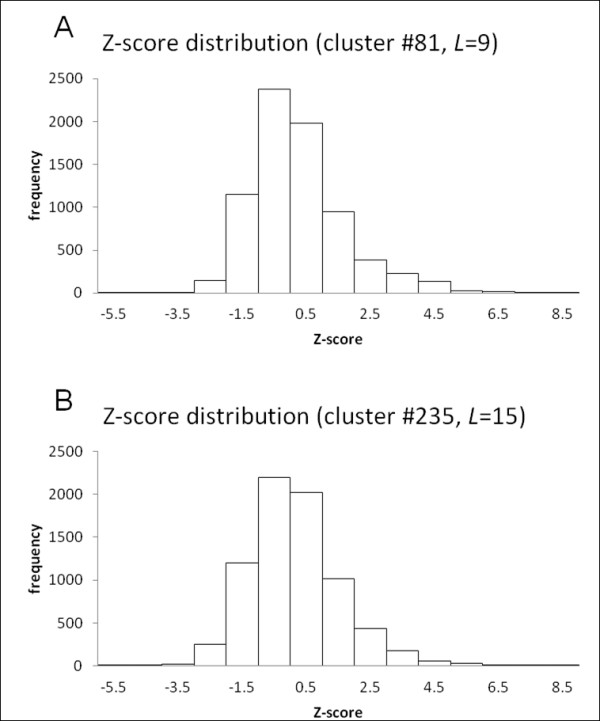
**Z-score distributions in cross profile analysis**. Two Z-score distributions of (A) cluster #81, as an example of for 9-residue-long segments, and (B) cluster #235, as an example of for 15-residue-long segments are shown.

Using FORTE, we compared the profiles of two different profile types: (i) a sequence-based profile stored in the FORTE library and produced by PSI-BLAST containing evolutionary information, and (ii) a structure-based profile (Figure 1). Structure-based profiles derived from local structural classification are expected to represent the protein structural information [16,19]. FORTE enables us to compare different profile types directly because it employs the correlation coefficient as a measure of similarity between two profile columns that are to be compared. We used structure-based profiles derived from clusters of segments as queries to find strong correlations with 7,419 sequence-based profiles in the FORTE library. Two examples of Z-score distributions of clusters for both 9-residue and 15-residue-long segments are shown in Figure 2.

We have analyzed structural clusters with at least 80 members to ensure that biases resulting from imperfect samples are avoided. Of 29,777 clusters for 9-residue-long segments, 449 had 80 members or more. Out of 80,254 clusters for 15-residue-long segments, 252 had 80 members or more. Of the 449 clusters for 9-residue-long segments, 12 clusters with *Z*-score of *(Z) *= 8 or higher were identified (Table 1), i.e., the 12 structure-based profiles of clusters showed significant correlation with 42 sequence-based profiles in the FORTE library for 9-residue-long segments. The threshold of the *Z*-score was determined empirically [22]. Conformations of medoid segments of the 12 clusters are presented in Additional file 1, Figure S1. Of the 252 clusters, 12 clusters with *Z *= 8 or higher were identified for the 15-residue-long segments (Table 2), i.e., the 12 structure-based profiles of clusters showed significant correlation with 50 sequence-based profiles. Conformations of medoid segments of the 12 clusters are shown in Additional file 1, Figure S2. As shown in both figures, the 24 clusters exhibit various conformations. Some are compact, although others are extended. These conformations consist of several secondary structure elements such as helices, strands, turns, and bulges. Neither a simple helix nor a simple strand exists. As might be expected, several similarities were observed among those profiles. For instance, the profile of cluster #81 in Table 1 was apparently similar to the parts of the profiles of clusters #148, #159, #164, and #235 in Table 2 because many members are common to those five clusters, i.e., many members of cluster #81 for 9-residue-long segments correspond to the parts of segments in clusters #148, #159, #164, and #235 for 15-residue-long segments, and many segments in cluster #148 were derived from adjacent positions of the segments in the cluster #159 (and others). Details of clusters #159 and #235 are discussed below (see (ii) 1jnrA:614-629 and 1kthA:16-31).

**Table 1:** Results of the cross profile analysis for 9-residue-long segments

Cluster ID (# of segments in the cluster)	Amino acid preferences	# of hits in the FORTE library	SCOP ID of hits	Average C_α_RMSD(Å)
81 (367)		3	g.8.1.1	0.49
140 (250)		1	a.118.8.1	0.96
181 (192)		1	a.118.8.1	2.81
184 (192)		4	a.118.8.1	0.30
232 (153)		1	d.37.1.1	4.25
239 (149)		3 1	g.41 i.1.1.2	0.44 1.54
246 (147)		8	a.118	0.81
247 (147)		3	a.118.8.1	0.32
313 (113)		1	a.118.8.1	0.85
366 (97)		14	a.39.1	0.92
375 (95)		1	b.34.7.1	1.99
438 (81)		1	g.3.11.1	1.94

**Table 2:** Results of the cross profile analysis for 15-residue-long segments

Cluster ID (# of segments in the cluster)	Amino acid preferences	# of hits in the FORTE library	SCOP ID of hits	Average C_α_RMSD(Å)
143 (126)		1	d.211.1.1	1.10
147 (124)		9	a.118	3.61
148 (124)		1	a.7.3.1	0.95
159 (119)		1 1	a.7.3.1 g.8.1.1	1.53 2.87
164 (113)		5	g.8.1.1	1.62
171 (109)		3	d.58	1.58
180 (105)		11	d.9.1.1	0.46
186 (102)		1	b.1.2.1	5.76
203 (97)		1	b.6.1.3	6.49
209 (92)		1 1	d.169.1.1 b.71.1.1	3.23 5.70
222 (89)		12	a.39.1	1.20
235 (84)		1 1	a.7.3.1 g.8.1.1	1.78 3.14

On average, C_α _RMSDs between the medoid segments of structural clusters and the segments of hits (*Z *≥ 8) in the FORTE library were, respectively, 0.84+/-0.89 Å for 9-residue-long segments, and 1.94+/-1.61Å for 15-residue-long segments. Although some exceptions with large RMSDs that might be false positives exist, these results are separate from the results of random match of 9-residue and 15-residue-long segments reported by Du *et al*. [23]. They calculated RMSDs between randomly chosen fragments and reported their distribution. They found that the centers of distributions for 9-residue and 15-residue-long segments were located, respectively, at 3.5 Å and 5.0 Å. Their definitions of segments with respect to the amount of secondary structures are matched with conformations of these segments (see Additional file 1, Figures S1 and S2). These results clearly indicate the structural similarity between conformations of a segment cluster and the local structure of a protein family. Generally, significant correlation between profiles of two different types indicates not only the similarities of amino acid substitution patterns but also those of the structural similarities of constituent segments of both sequence-based and structure-based profiles.

The 12 profiles derived from the structural clusters for 9-residue-long segments showed correlation with sequence profiles in seven different protein folds according to the SCOP classification. Half of them showed correlation with 18 sequence profiles of segments in proteins that possess an α-α superhelix fold (SCOP ID: a.118). In Table 1 the profile of cluster #181 was apparently similar to the profiles of clusters #184, #246, and #247. These were the 'adjacent-segment' effects described above. Similarly, the profile of cluster #140 was similar to that of cluster #313 in Table 1 (and also to that of #147 in Table 2). The profile derived from cluster #366 showed strong correlation with 14 sequence profiles of segments corresponding to Ca^2+^-coordinating loops in proteins of the EF-hand superfamily (SCOP ID: a.39.1). The 12 clusters of 15-residue-long segments show correlation with a more diverse set of proteins (Table 2) than was the case for the clusters of 9-residue-long segments, i.e., correlation observed in 11 different protein folds. However, most of the correlations above the threshold were observed between the sequence profiles of segments of the EF-hand superfamily and the profiles derived from cluster #222, which clearly reflects the functional constraints on protein sequence evolution. Apparently, the profile of cluster #366 in Table 1 corresponds to part of the profile of clusters #222 in Table 2.

In principle, methods used for the structural classification of the protein segments are expected to affect structure-based profiles. However, a small change of parameters such as a threshold variable for structural similarity *D*_th _used for clustering has been demonstrated not to have much effect on the results in our previous study [16]. We observed robustness of the shapes of the distribution of segment clusters. For instance, we showed the dependence of a threshold parameter on the clustering results is minimum around *D*_th _= 30°, which we used for this study, to 40° (see [16] for more details).

### Preserved sequence-structure patterns

In the cross profile analysis of the 15-residue-long segments, we identified preserved sequence-structure patterns that transcend protein superfamily or fold boundaries that were previously undetectable (cf. Table 2).

#### (i) 1p1lA:2-16, 1kr4A:7-21, and 1mwqA:58-72

**Figure 3:**
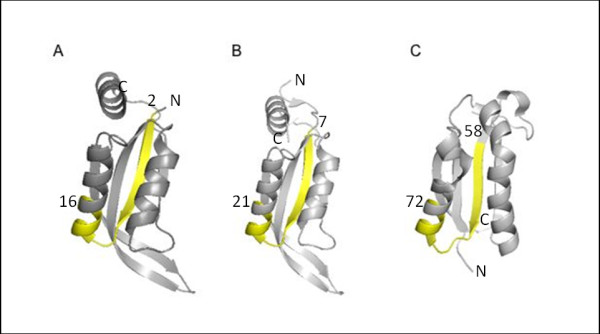
**Structures of the preserved segments in ferredoxin-like fold proteins**. Three ferredoxin-like fold proteins are shown. The corresponding portions of (A) 1p1lA:2-16, (B) 1kr4A:7-21, and (C) 1mwqA:58-72 are in yellow.

The structure-based profile of cluster #171 of 15-residue-long segments showed significant correlation (*Z *≥ 8; see above) with the three sequence profiles of 1p1lA:2-16 (Figure 3A), 1kr4A:7-21 (Figure 3B), and 1mwqA:58-72 (Figure 3C). According to the SCOP classification, these three proteins belong to the ferredoxin-like fold (SCOP ID: d.58) category. Two of them, 1p1lA and 1kr4A are members of the same CutA1 family in the GlnB-like superfamily, whereas 1mwqA belongs to the YciI-like family in the dimeric α+β barrel superfamily. In the CATH database, the three proteins possess the same α-β plaits topology (CATH ID: 3.30.70); 1p1lA and 1kr4A are classified as having CATH ID: 3.30.70.830 topology, and 1mwqA is classified as a dimeric α+β plaits protein (CATH ID: 3.30.70.1060). The ferredoxin-like fold, one of the SCOP superfolds, consists of two repetitive βαβ units. It is particularly interesting that the sequence profiles of the structurally corresponding regions, the N-terminal half of the first βαβ unit in 1p1lA and 1kr4A, and the N-terminal half of the second βαβ unit in 1mwqA, showed significant correlation with the same profile cluster #171, in spite of the differences in their sequential positions (Figure 3). This result might indicate that structure actually shapes sequence evolution or it might result from context (or environment)-dependent substitutions of amino acids. Alternatively, the correlation might be a relic of the duplication of a βαβ unit in the evolution of proteins with the ferredoxin-like fold [24].

#### (ii) 1jnrA:614-629 and 1kthA:16-31

**Figure 4:**
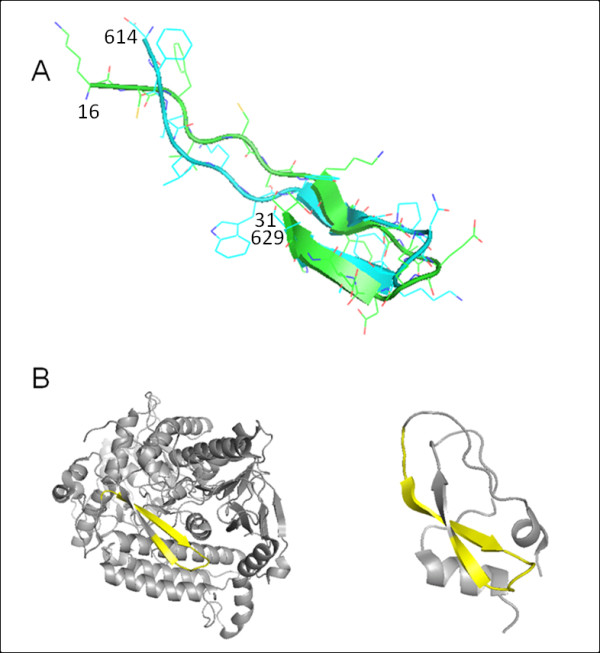
**Structural superposition of the two preserved segments in two unrelated proteins with different folds**. (A) Two β-hairpin-like segments of FLVC-segment (green) and BPTI-segment (blue) are superimposed (2.49Å C_α _RMSD). (B) Different structures of 1jnrA (left) and 1kthA (right) are shown. The corresponding portion (yellow) of the two segments forms a β-hairpin-like structure in both proteins.

We were unable to recognize the evolutionary relations between the two proteins, chain A of 1jnr and chain A of 1kth. However, two segments of 1jnrA:614-629 (hereinafter FLVC-segment) and 1kthA:16-31 (hereinafter BPTI-segment) form similar conformations (Figure 4A) in two unrelated proteins with different folds (Figure 4B); 1jnrA is the α-subunit of adenylylsulfate reductase that reversibly catalyzes the reduction of adenosine 5'-phosphosulfate to sulfite and AMP [25], and 1kthA is a protease inhibitor that corresponds to the C-terminal Kunitz-type domain from the α3 chain of human type VI collagen [26]. Based on SCOP 1.73 release [27], the FLVC-segment is embedded in domain 1 (503-643), which is in the spectrin repeat-like fold class (SCOP ID: a.7). The BPTI-segment is categorized in the BPTI-like fold class (SCOP ID: g.8). Domains that contain the spectrin repeat-like fold usually comprise three α-helices [28,29]. However, the entire fold of 1jnrA is classified as the disulfide-rich α+β fold. In addition, according to the CATH classification [30], most of the 1jnrA fold is in the domain that possesses the FAD/NAD(P)-binding domain topology (CATH ID: 3.50.50.60). 1kthA is categorized into the factor Xa Inhibitor topology (CATH ID: 4.10.410).

In both 1jnrA and 1kthA, the sequence profiles of two consecutive 15-residue length segments show significant correlation (*Z *≥ 8) with structure-based profiles of two clusters (Table 2). The N-terminal regions of 1jnrA:614-628 and 1kthA:16-30 showed correlation with cluster #235, whereas the C-terminal regions, 1jnrA:615-629 and 1kthA:17-31 showed correlation with cluster #159. The structure-based profiles reflect the results from the structural classifications of the protein segments. Therefore, we investigated the composition of the two clusters #235 and #159 to check whether segments similar to those of 1jnrA and 1kthA are included in them. Most of the segments in the two clusters mutually overlap. As expected, 61 out of the 84 segments in cluster #235 and 119 segments in cluster #159 are derived from adjacent positions in the same proteins. The clusters contain segments that mainly originate from all-β (ca. 40%) and α+β proteins (ca. 27%). However, it is unlikely that this suggests bias in the usage of the folds because the segments are derived from 58 folds (cluster #235) and 76 folds (cluster #159). Although the two proteins, 1g6x and 2knt, from the BPTI-like fold class (SCOP ID: g.8) are included in the clusters, no protein of the spectrin repeat-like fold class (SCOP ID: a.7) is incorporated. Consequently, at least for 1jnrA, no readily apparent evolutionary relation exists to explain the remarkable correlation between sequence-based and structure-based profiles. The segments of the two structural clusters are included in Additional file 2, Table S1.

**Figure 5:**
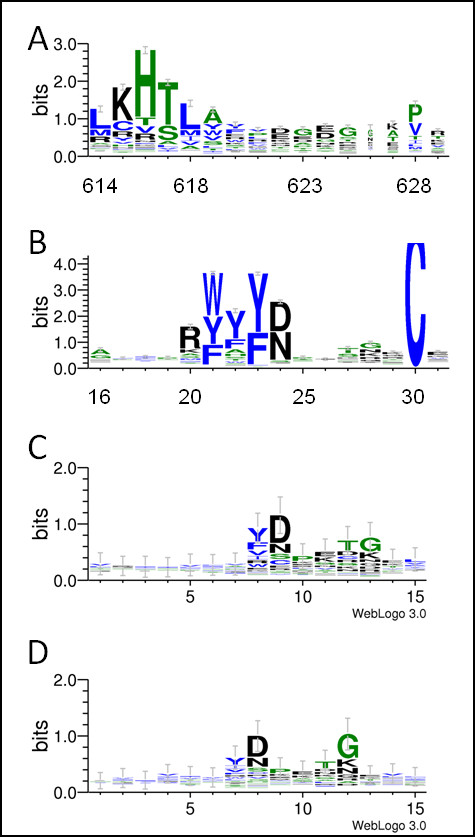
**Graphical representation of sequence conservation patterns**. Sequence conservation patterns of the corresponding regions of the profiles of (A) FLVC-segment, (B) BPTI-segment, (C) cluster #235, and (D) cluster #159 were drawn using WebLogo 3 [62].

Similar patterns of sequence conservation between the sequence profiles of the FLVC-segment and the structure-based profiles of clusters #235 and #159 are readily identifiable. Figure 5 shows the sequence conservation patterns of the corresponding regions of 1jnrA:614-629 (in the Pfam [31] protein family PF02910) and of 1kthA:16-31 (in PF00014), and the corresponding regions of clusters #235 and #159. Although we observed family-specific residue conservation in each sequence profile, we also found that the Tyr and Asp residues at the eighth and ninth positions of the regions corresponding to the FLVC-segment and BPTI-segment were conserved. This corresponds to the structural clusters in which the eighth and ninth positions of cluster #235 and the seventh and eighth positions of cluster #159 are conserved. Furthermore, the conserved Gly residue at the 13^th ^position of the regions corresponding to the FLVC-segment and BPTI-segment is also conserved at the 13^th ^position in cluster #235 and at the 12^th ^position of cluster #159. These conserved residues are located close to the turn region of β-hairpin-like structures. The conservation patterns of residues near the turn region of the segments discussed above resemble *chignolin*, the short peptide which spontaneously folds in water [32].

Our classification results obtained using the SCOP 1.73 release (November 2007) show that there are 15 superfamilies with the spectrin repeat-like fold among the clusters. Of those, domain 1 of 1jnrA:503-643 contains the 1jnrA:614-629 segment belonging to the succinate dehydrogenase/fumarate reductase flavoprotein C-terminal domain superfamily. Of the 15 superfamilies, only three, succinate dehydrogenase/fumarate reductase flavoprotein C-terminal domain, ribosomal protein S20, and PhoU-like superfamilies, have an 'additional' β-sheet at the C-terminus portions. Compared to the β-sheet of 1jnr, the region corresponding to both the β-sheet at the C-terminus portion of ribosomal protein S20 and the PhoU-like superfamily is small. Moreover, according to SCOP, the region is assigned to other domains that belong to other folds, instead of to the spectrin repeat-like fold, as is true when other classification databases such as CATH and VAST [33] are used. According to the classification of both the CATH and SCOP database, the BPTI-like fold (or the factor Xa Inhibitor topology) consists of a single superfamily.

### Sequence evolution of the segments in each family

We measured the 'direction' of the amino acid sequence evolution of the segments, including the FLVC-segment and BPTI-segment, as described above, in terms of the compatibility with the structure-based profiles. This compatibility might reflect the physicochemical constraints or preferences of segment conformations in clusters #235 and #159. We calculated the score *S *for a sequence in the structure-based profiles of clusters #235 and #159 (see eq. (2) in Methods), and postulated that high scores indicate high compatibility of the sequence with the profile. We compared the scores between existing and deduced ancestral sequences, and considered that differences in the scores *ΔS *(see eq. (3) in Methods) reflect the direction of sequence evolution. Here, the results suggest that negative *ΔS *means that existing sequences are less compatible with the structure-based profile than their ancestral sequences in terms of β-hairpin-like structure that we identified.

**Figure 6:**
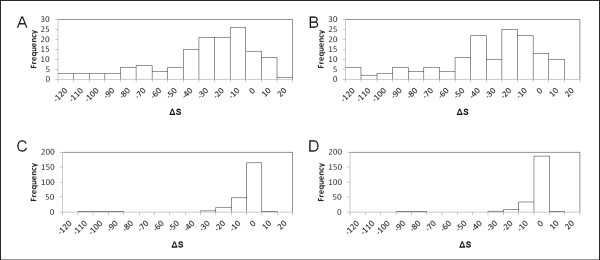
**Distribution of score differences between the ancestral and existing sequences**. The score differences *ΔS *(deltaS) between the ancestral and existing sequences of two protein families are shown: *ΔS *of the PF02910 sequences for the structure-based profiles of clusters (A) #235 and (b) #159, and *ΔS *of the PF00014 sequences for the structure-based profiles of clusters (C) #235 and (d) #159.

**Figure 7:**
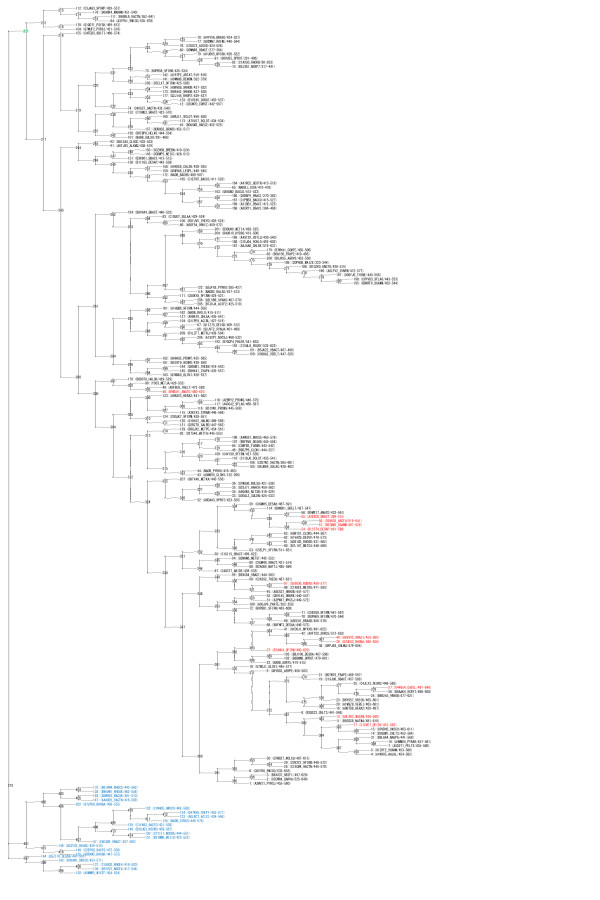
**Phylogenetic tree of the PF02910 family sequences**. The phylogenetic tree of 40% representatives of PF0291 and 1jnrA (= O28603_ARCFU/519-641) generated by ANCESCON is shown. Proteins with positive values of *ΔS *are shown in red. 22 sequences that were excluded from the calculation are shown in blue. The next root position regarded as an ancestral protein is shown in green.

We identified the commonalities and differences between the two protein families. The range of score distributions of existing sequences (from around -20 to 10), except for those with gaps based on the Pfam alignments, was almost always the same. In contrast, the deduced ancestral sequences of the two families have different scores. The scores for the ancestral sequence of the Pfam protein family ID: PF02910 are, respectively, 0.28 for the profile of cluster #235, and 2.87 for the profile of cluster #159. Meanwhile, the scores of ancestral sequence of the PF00014 family are 11.00 for cluster #235, and 11.04 for cluster #159. Therefore, the score differences *ΔS *between the ancestral and existing sequences of the two protein families show different distributions (Figure 6). Substantial portions of *ΔS *are distributed from around 0 to -40 in both families. However, some existing proteins of PF02910 give positive values for *ΔS*, although all except one of the existing sequences of PF00014 give negative values for *ΔS*. This result suggests that the sequences of several subfamilies, including 1jnrA of PF02910, have evolved towards increased compatibility with the structure-based profiles (Figure 7), which seems to indicate that a convergent evolution might have occurred at the corresponding region of 1jnrA(:614-629) and its subfamily.

**Figure 8:**
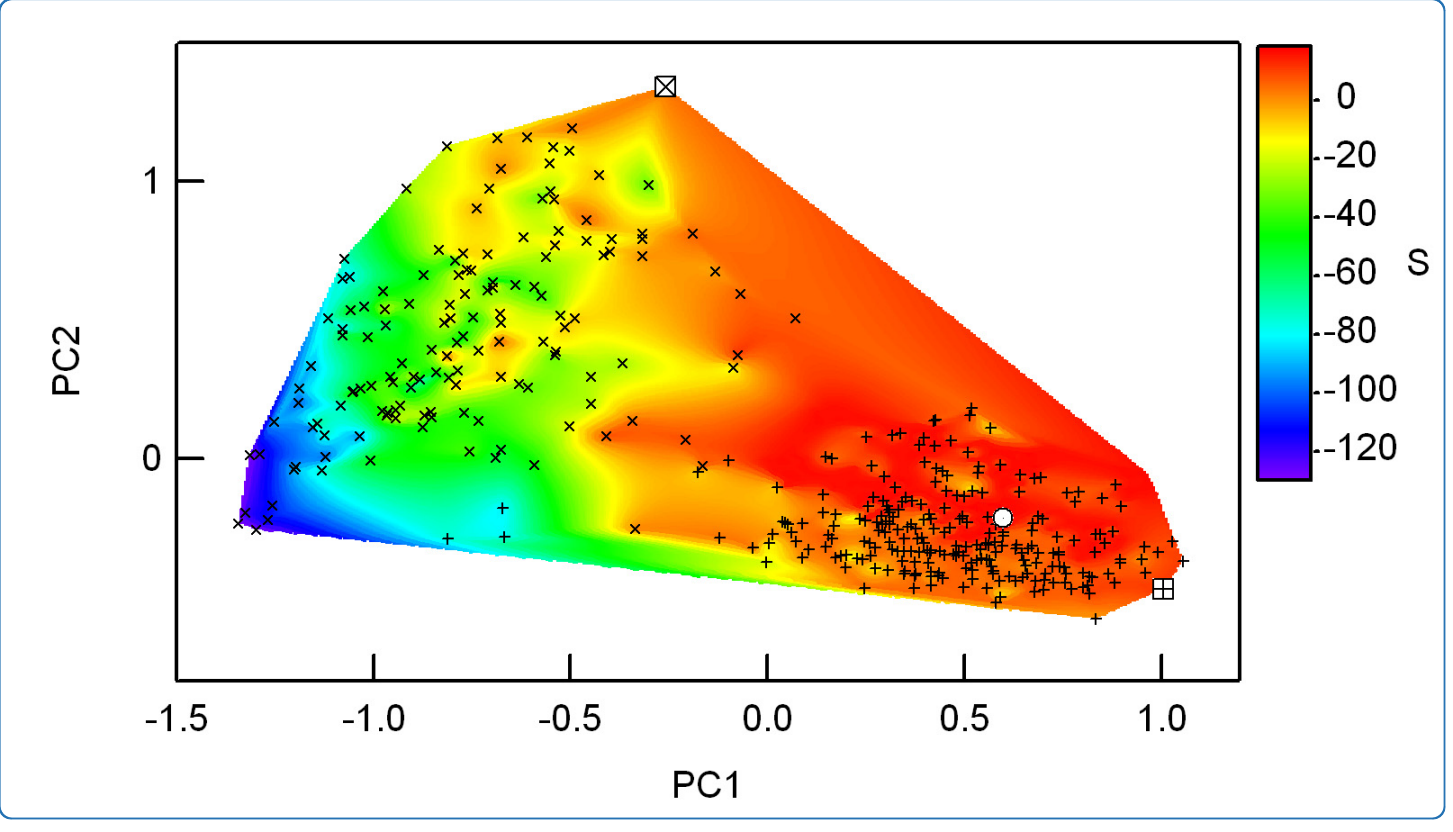
**Schematic representation of an evolutionary landscape of the segments**. The contour map in a sequence space represents compatibility with the structure-based profile of the β-hairpin-like structure we identified. Points closer to the highest point (open circle) on the map are more compatible with the structure-based profile of cluster #159. Crosses and inclined crosses represent segments in presently existing proteins, which are classified respectively into two families, PF00014 and PF02910. Squares indicate ancestral sequences of each family, so that the map involves evolutionary directions of present segments from their ancestors. The sequence space in the map is defined by the PCA axes (PC1 and PC2). These axes were determined using principal component analysis (PCA) of sequences of all segments, in which the Hamming distance was used as a dissimilarity parameter between the two sequences. Contour levels shown with color scaling were drawn by the interpolation algorithm embedded in IGOR using the compatibility values *S *of both existing and virtual sequences.

Figure 8 presents an evolutionary landscape in which a contour map shows compatibility with the structure-based profile of cluster #159, β-hairpin-like structures. Segment sequences of the PF02910 and PF00014 families were projected onto a XY-plane, which represents a sequence space (see the legend of Figure 8). The higher the point in the map, the greater is the compatibility with the structure-based profile. Two ancestral sequences, indicated by squares on the map, are distant from one other, implying polyphyletic evolution. Once two distinct ancestral sequence segments with similar β-hairpin-like structures had emerged, the segments of both families evolved within certain areas in the sequence space. Wide sequence divergence of the segment of PF02910 (inclined crosses) is shown in the map because they would be free from functional or physicochemical constraints. Sometimes large deletion(s) occurred in their sequences, according to the multiple alignment provided by PFAM. Apparently some PF02910 sequences with positive *ΔS*, i.e. more stable than their ancestral sequences, evolved in the direction of PF00014 (crosses) and/or the highest, i.e. the most stable point (open circle) on the map. The sequence distribution of the segment of PF00014 is limited around the highest point, probably because of the role of segment stability, which is expected to be more important for small proteins such as those of the PF00014 family.

The results might be explainable using either of two evolutionary scenarios: divergent or convergent evolution. However, for the following reasons, we speculate that those segments originated from distinct ancestors in this case. First, we found similarities between the structure-based profiles and the sequence profiles of two distinct protein families rather than direct similarities between segments of two distinct families. Consequently, it is difficult to hypothesize that those segments originated from a common ancestor through an evolutionary mechanism that necessarily occurred before the divergence into two distinct families. Although sequences of the Pfam protein family ID: PF02910 are distributed mainly in bacteria, most sequences in the Pfam protein family ID: PF00014 are distributed in eukaryotes. In addition, the functions and localization of two protein families are completely different. Protein sequences of PF02910 are parts of reductases, dehydrogenases, and oxidases in a cell. In contrast, proteins of PF00014 are secreted proteins which function as protease inhibitors or toxins. Furthermore, for example, in humans, 1kthA (= CO6A3_HUMAN/3111-3163) is encoded in an exon, i.e. no exon boundaries exist in its portion. There are no introns in the gene that encodes 1jnrA (= O28603_ARCFU/519-641), which is a portion of a large archaeal protein. Finally, it is difficult to imagine that present proteins of PF00014 were derived originally from both the turn region of β-hairpin-like structures and the rest because these proteins are too small to be stable and functional without this region. Taken together, the similarity between segments presented here does not necessarily indicate common evolutionary ancestry. It is apparently a reflection of physicochemical constraints of local conformations, i.e., it seems probable that convergent evolution might have occurred for this case. The evolutionary directions analyzed in Figure 8 also support the scenario of convergent evolution.

### Implications for short autonomous elements

We have identified several structural clusters with structure-based profiles that show remarkably strong correlation with sequence-based profiles. We have observed that most segments are structurally similar, and are similar also to other segments in the cluster(s). For example, 15-residue-long segments of 1jnrA:615-629 in the FLVC-segment and 1kthA:17-31 in the BPTI-segment are similar to one another. The two segments are also similar to segments in cluster #159, whose profile indicates significant correlation with their sequence-based profiles. Do segments fold into particular structures irrespective of their context? To ascertain this, we synthesized 15-residue peptides with the deduced sequence of cluster #159 (TIIMWYYDPETGEWW), which has the highest score, i.e. the most compatible sequence with the structure-based profile of cluster #159, and conducted several experiments to elucidate its 3D-structure in aqueous solution.

**Figure 9:**
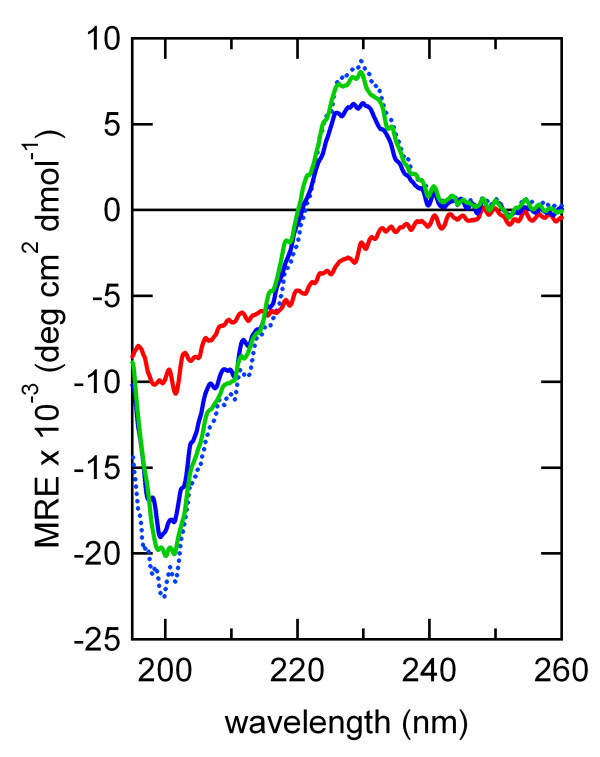
**Far-UV CD spectra of the consensus peptide of cluster #159**. CD spectra of the 15-residue peptide with the consensus amino acid sequence, TIIMWYYDPETGEWW, of cluster #159 are shown. The CD spectra of the peptide at 20°C (293 K, blue line) and 5°C (278 K, green line) are similar. Temperature-dependent spectra show thermal denaturation at 98°C (371 K, red line) and renaturation at 20°C (293 K, dotted blue line) after the temperature-jump from 98°C (371 K) of the peptide.

Conformational analysis of the synthetic peptide by circular dichroism (CD) spectroscopy revealed that the peptide had an autonomous element that exhibited high foldability and stability. The far-UV CD spectra of the peptide at 20°C (293 K) and 5°C (278 K) show a characteristic positive peak at 229 nm, which is probably attributable to an edge-to-face exciton couplet between Tyr and Trp [34-36], which suggests that the peptide forms a β-hairpin-like structure resembling the corresponding portion of the elements FLVC-segment and BPTI-segment and the segments in cluster #159 that we found in their native states. We also observed reversible thermal refolding when we cooled the peptide solution from 98°C (371 K) to 20°C (293 K) (Figure 9). As we noted above, the residue conservation patterns in the turn region resembles that of *chignolin*. We therefore suggest that the residues around the turn region might be important to confer high foldability and stability to the peptide. Consequently, these results strongly suggest that the peptide folds autonomously into a unique structure in aqueous solution, and further indicates that segments with sequences similar to the profile of cluster #159 probably fold into the same local structure independent of the context (i.e. in any folds). This is true even when no evolutionary relation exists between the folds. These results suggest that the structure-based profiles represented by these clusters reflect the physicochemical preferences of ancient short peptide ancestors. They also suggest the role of the segments as structural building blocks, and the existence of ancient short peptide ancestors.

Such speculation can be inferred not only from our results but also from other experimental studies. The peptide described above is not a first short autonomous element, derived from native proteins, that exhibits high foldability and stability. Several short fragments such as C-peptide of ribonuclease A [37], a C-terminal helix of cytochrome c [38], G-peptide of protein G [39,40] and an N-terminal fragment of ubiquitin [41] forms their native-like conformations by themselves, although most isolated fragments cannot retain the original conformation without interactions with the remaining proteins. In addition, several pioneering works have succeeded in creating artificial assemblies that consist of a combination of short fragments as structural building blocks [42-48].

## Conclusions

In 9-residue-long and 15-residue-long segments, we identified several segment clusters with structure-based profiles that show significant correlations (*Z *≥ 8) with sequence-based profiles. We found significant correlation between a sequence-based profile and a structure-based profile, indicating structural similarity between the local structure of a protein family and representatives of a segment cluster. We found exceptionally strong correlation between amino acid preferences and local structures in all except one of the 42 9-residue-long segments (*L *= 9) and in 47 of the 50 15-residue-long segments (*L *= 15). These results suggest strong correlation between sequence substitution patterns and structures for some elements in proteins, in agreement with earlier results [13,49]. Results also suggest that our method does not require calculation of the structural similarity between two segments to identify similar segments in both sequence and structure, in contrast to previous studies [1,7].

Although many examples of significant correlations between sequence profiles and structural profiles of protein segments are apparently related to divergent evolution, several sequence-structure patterns that transcend protein family, superfamily, and even fold boundaries were identified. In those cases, the patterns found in the ferredoxin-like fold correspond to structurally equivalent segments within the fold. This example suggests the duplication of ancestral segments.

Through cross profile analysis, this report elucidates the preserved sequence-structure patterns, which designate β-hairpin-like structures shared by different protein folds. Based on the evolutionary analysis of two distinct proteins, these segments might be examples of convergent evolution using the sequence and structural information of consecutive segments. These results present a clear contrast to those of an earlier study [9] which found exclusively distant evolutionary relations using an order-independent profile-profile method. Most examples reported in the present study are apparently not under functional constraints, except for the EF-hand motif. In general, sequence-function correlations such as the catalytic triads and the EF-hand motif are often prominent and are easier to detect than sequence-structure correlations. Our cross profile analysis method is able to detect subtle sequence-structure correlation.

Irrespective of residue environments in proteins, these segments whose sequence-based profiles show correlation with structure-based profiles of specific clusters (#159 and #235) have well-preserved structures. Therefore, we examined the conformational properties, in aqueous solution, of a consensus peptide sequence from a cluster with these properties. CD spectral analysis of the peptide solution strongly suggests that the peptide has the property of a short autonomous element that exhibits high foldability and stability. This observation suggests that segments of the clusters that show good correlations with sequence-based profiles are autonomous elements, which are also local sequence/structure motifs, such as those in the I-sites library [13]. Other reports have described the potential use of local sequence information to improve protein structure prediction. This report describes a new water-soluble β-hairpin-like peptide, which might support the hypothesis of polyphyletic origins of presently existing protein domains. Lupas *et al*. [1] discussed the possibility of the evolution of proteins from peptides and argued that one candidate ancient peptides or fundamental elements of proteins is a β-hairpin-like peptide [24]. The results presented here provide new insights into the evolution of protein short segments. Moreover, they are expected to be useful in improving our understanding of protein folding and evolutionary mechanisms.

## Methods

### Construction of profile libraries

#### Preparation of structure-based profiles

The local structures of 9-residue-long and 15-residue-long protein segments were classified to obtain structure-based profiles. A non-redundant dataset of protein structures was used for classification. Representative proteins were obtained from the PDB select dataset (Sep. 25, 2001, version) [50], which contains 1,614 chains (resolution < 3.0 Å; R-factor < 0.3; sequence identity < 25%). Representative proteins were divided into short segments using a sliding *L*-residue window. Segments can be mutually overlapping.

Local structures of segments consisting of consecutive *L *(= 9, 15) amino acids were classified using a single-pass clustering method [51] as follows: i) Choose a segment and declare it to be in a cluster of size one. ii) Choose the next segment and compute distances from this segment to the centroids of all clusters. iii) Add the segment to the "nearest" cluster. If no cluster is sufficiently close (within a certain threshold), then declare the segment to be in a new cluster. In step iv) Go back to (ii) and repeat the process until all segments are classified. All parameters characterizing the distribution of the local structures were determined directly by assigning an arbitrary value to a threshold variable for structural similarity, *D*_th_, that is defined based on the backbone dihedral angles. In this study, clustering results were obtained by assigning 30° to *D*_th_. Detailed explanations of the clustering method can be found in a related paper [16].

Profiles showing the statistical propensities of amino acids of segments in a certain cluster were calculated from the observations of amino acid occurrences at each position within a segment cluster. The matrix was prepared by scoring a multiple alignment of sequences. Structure-based profiles, whose element is *pro_i_*(*j*), of amino acid *j *at position *i *in a cluster are defined as shown below.

(1) $$pro_{i}\left(j\right)=p_{i}\left(j\right)∕p\left(j\right)$$

In that equation, *p_i_*(*j*) represents the probability of observing amino acid *j *at position *i *in the segments of a cluster, and *p*(*j*) signifies the composition of amino acid *j*. Although several methods exist to convert a multiple alignment into a score, we employed a simple amino acid propensity that was calculated with neither weights nor pseudo-counts for this study. This propensity corresponds to the ratio of the frequency count of a certain residue type appearing at a particular position to the global frequency count of the amino acid residues. The segments and information of amino acid preferences in each structural class were classified using ProSeg: a database of local structures of protein segments http://riodb.ibase.aist.go.jp/proseg/index.html[52].

#### Preparation of sequence profiles

The FORTE system (see below) holds the sequence profile library of representative proteins whose structures are known. The amino acid sequences of those proteins are derived mainly from the ASTRAL [53] 40% identity list according to the SCOP classification [27]. Representative sequences that are not in SCOP were selected from the PDB entries [54]. The FORTE library includes 7,419 sequence-based profiles.

To generate the sequence PSSMs of the library, PSI-BLAST iterations with the nonredundant (NR) amino acid sequence database from NCBI [55] were performed up to 20 times. The NR NCBI protein database was clustered using a 95% sequence identity threshold and the CD-HIT program [56] to reduce computational time. The 95% representative sequences of the NR NCBI protein database were then masked using the *pfilt *program in the PSIPRED package [57]. When we performed PSI-BLAST iterations, we set 5 × 10^-4 ^as the e-value cutoff value for inclusion in the next pass [58]. We applied the *makemat *program of the IMPALA package [59] to prepare the PSSMs from the PSI-BLAST outputs.

### Profile-profile comparisons

We have developed our own profile-profile comparison method, the Fold Recognition Technique (FORTE), which uses large amounts of sequence information, optimized gap penalties, and correlation coefficients as the scoring scheme to measure the similarity between two profile columns. Using FORTE, profile-profile comparisons were performed. To build an optimal alignment between two compared profiles, we used the global-local algorithm, which is based on the global alignment algorithm with no penalty for the terminal gaps. The significance of each alignment score is estimated by calculating *Z*-scores using a simple log-length correction. The FORTE server is available at http://www.cbrc.jp/forte/[21]. Successful examples of its application can be found in the literature [11,22,60]. For the present study, we used position-specific matrices derived from local structural classifications as query PSSMs to find significant correlation with sequence profiles (Figure 1).

### Score calculation of ancestral and existing sequences for a profile

#### Construction of ancestral sequences

To obtain the ancestral sequences of the two Pfam protein families, PF02910 and PF00014, we used the set of 40% representative sequences clustered by the CD-HIT program with 'full' members of the Pfam families (3,109 PF02910 sequences and 2,143 PF00014 sequences), and by adding 1jnrA (= O28603_ARCFU/519-641) to the 40% representative PF02910 sequences and 1kthA (= CO6A3_HUMAN/3111-3163) to the 40% representative PF00014 sequences. The root sequences were generated by ANCESCON [61] with the "Alignment-Based rate factor" method based on the Pfam alignments of selected sequences (209 sequences from PF02910 and 236 sequences from PF00014) described above. For the PF02910 family, we regarded the next root sequence (see Figure 7) as an ancestral sequence because the deduced root sequence lacked two amino acids in the segment that corresponds to the FLVC-segment. One branch comprising 22 sequences that lack most amino acids in the region of interest was excluded from the following calculation.

#### Calculation of scores for a structure-based profile

We calculated the sum of log-odds scores, *S *for both ancestral and existing sequences for a structure-based profile to elucidate the direction of sequence evolution in terms of the compatibility with the structure-based profile.

(2) $$S=\overset{L}{\underset{i=1}{\sum }}\ln\left(pro_{i}\left(j\right)\right)$$

In that equation, *L *(= 15) represents the length of a structure-based profile. Please see eq. (1) for *pro_i_*(*j*). In this calculation, we used -9.21 (≈ln(0.0001)) as the penalty for a gap, and also used the same value for the *p_i_*(*j*) = 0 case to avoid undefined values of the logarithm. The score differences *ΔS *between the ancestral (*S*_r_) and existing sequences (*S*_ℓ_) are also calculated as shown below.

(3) $$\Delta S=S_{\ell }-S_{r}$$

In this calculation, of the 187 representative PF02910 sequences, we excluded 43 sequences that have no amino acids in the segment that corresponds to the FLVC-segment.

### Peptide preparation

The synthetic peptide (TIIMWYYDPETGEWW) was purchased from Biosynthesis Inc. (Lewisville, Texas, USA). The identity and purity of the peptide were confirmed using mass spectrometry with a MALDI-TOF MS instrument (Voyager; Applied Biosystems) and using reversed-phase chromatography with an AKTA purifier (GE Healthcare) and a C18 column. Both the N-terminal and C-terminal of the peptide were in free-form (not protected).

### Peptide conformation analysis

Circular dichroism (CD) spectra were recorded on a J-805 spectropolarimeter. The synthetic peptide was dissolved at 0.26 mM in 70 mM sodium phosphate buffer (pH 8.0). Spectra were measured at several temperatures and represented in units of molecular ellipticity per mole of residue (MRE). Thermal denaturation of the peptide was almost reversible (ca. 100%), as judged by recovery of the spectra upon cooling.

## Authors' contributions

KT conducted most of the calculations necessary for this study, and wrote the manuscript. YS prepared structure-based profiles and the evolutionary landscape. SH conducted laboratory experiments, and helped with the design of the study and writing. All authors read and approved the final manuscript.

## Supplementary Material

Additional file 1 **Figure S1. Medoid segments of the 12 clusters in Table **1. Conformations of medoid segments in ProSeg for the 12 clusters in Table 1 (*L *= 9) are shown. **Figure S2. Medoid segments of the clusters in Table **2. Conformations of medoid segments in ProSeg for the 12 clusters in Table 2 (*L *= 15) are shown. Click here for file

Additional file 2 **Table S1. Members of the two clusters (#235 and #159) in ProSeg**. The segments of the two clusters (#235 and #159) are listed. The IDs, start positions, PDB IDs, chain IDs, and sequences of the segments in the ProSeg database are shown. Click here for file
