# Supplementary material for: Convergent evolution in structural elements of proteins investigated using cross profile analysis
Source: BMC Bioinformatics. 2012 Jan 16;13:11. doi: 10.1186/1471-2105-13-11 (PMC3398312; doi:10.1186/1471-2105-13-11)
Supplement: Additional file 1 — Figure S1. Medoid segments of the 12 clusters in Table 1. Conformations of medoid segments in ProSeg for the 12 clusters in Table 1 (L = 9) are shown. Figure S2. Medoid segments of the clusters in Table 2. Conformations of medoid segments in ProSeg for the 12 clusters in Table 2 (L = 15) are shown. [file 1471-2105-13-11-S1.PDF]

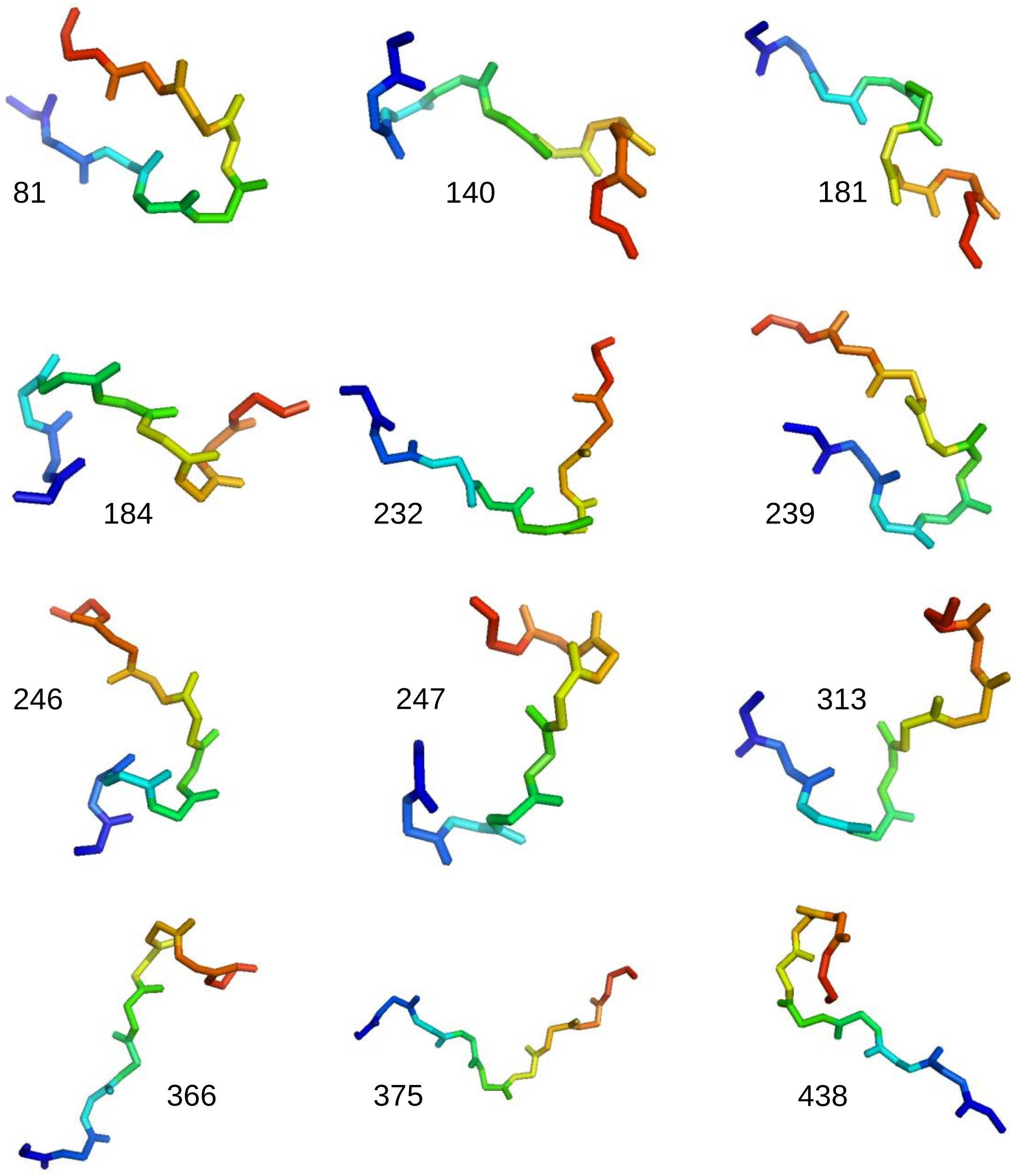

Figure S1

Conformations of medoid segments of the 12 clusters in Table 1 (*L*=9)

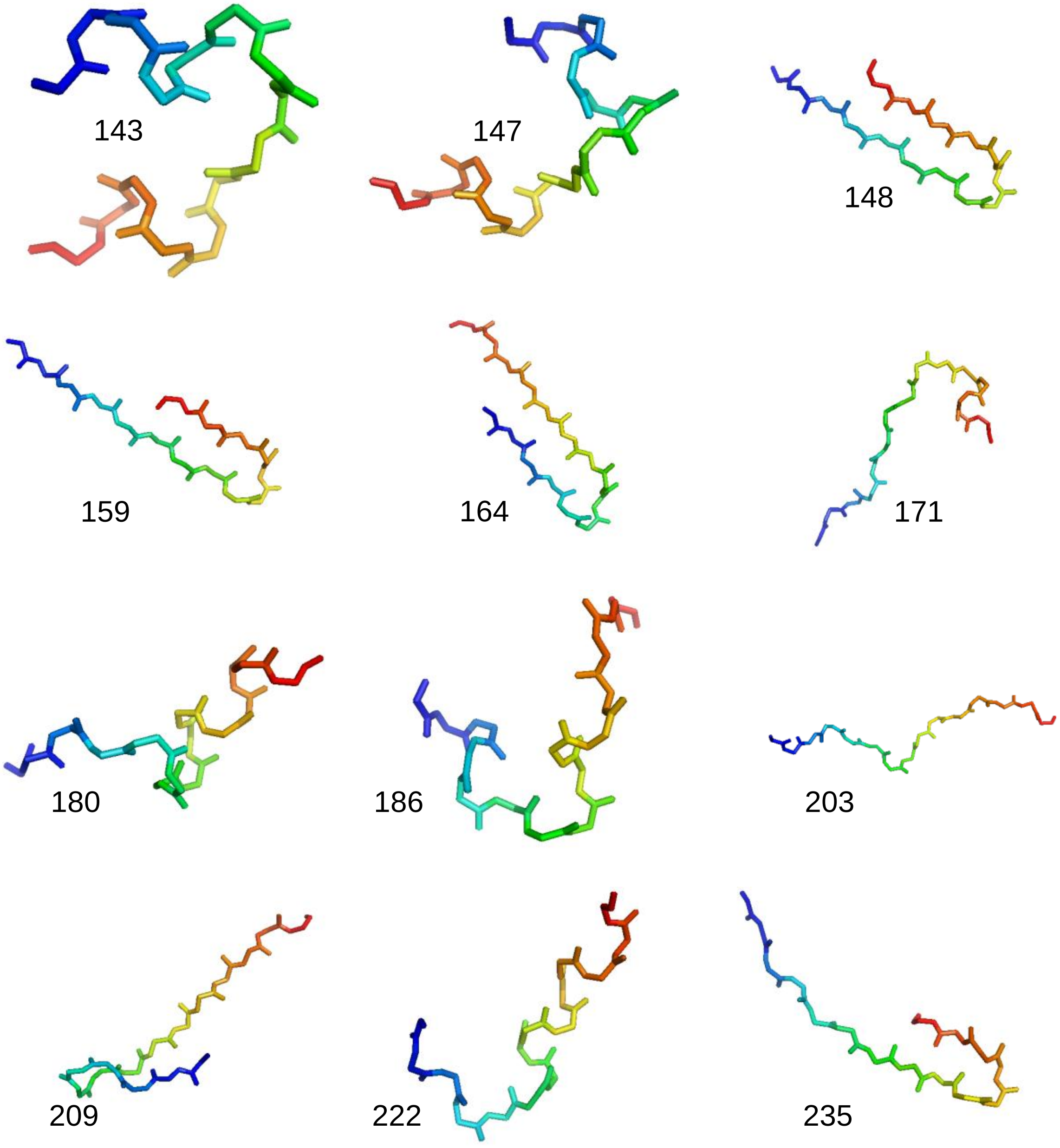

Figure S2

Conformations of medoid segments of the 12 clusters in Table 2 (*L*=15)
